# Standardization and Quality Control of the Herbal Medicine Mist Nibima, Employed to Treat Malaria and COVID-19, Using Physicochemical and Organoleptic Parameters and Quantification of Chemical Markers via UHPLC-MS/MS

**DOI:** 10.1155/2021/6390481

**Published:** 2021-12-01

**Authors:** Emmanuel Kofi Kumatia, Francis Ofosu-Koranteng, Alfred Ampomah Appiah, Kofi Bobi Barimah

**Affiliations:** ^1^Department of Phytochemistry, Centre for Plant Medicine Research, Mampong-Akuapim, Ghana; ^2^Department of Food and Agriculture, Ghana Standard Authority, Accra, Ghana; ^3^Department of Clinical Research, Centre for Plant Medicine Research, Mampong-Akuapim, Ghana

## Abstract

Mist Nibima is an essential herbal medicine used to treat malaria, bacterial, yeast, and COVID-19 infections. However, the drug has not been standardized and its active chemical ingredients are also not known. This study employed physicochemical, organoleptic, qualitative, and quantitate phytochemical analysis to established standards for Mist Nibima. Additionally, UHPLC was used to quantify the alkaloid cryptolepine in the drug using calibration curve. The chemical ingredients in Mist Nibima were thereafter characterized using UHPLC-MS. Organoleptic evaluation shows that Mist Nibima is a very bitter, cloudy, broom yellow decoction with the following physicochemical parameters: pH = 6.10 ± 0.08 (at 28.3°C), total solid residue = 5.34 ± 0.27%w/v, and specific gravity = 1.0099 ± 0.0000. The total alkaloid (23.71 ± 1.311%) content of the drug is 3 times its total saponins (7.993 ± 0.067%) content. Methyl cryptolepinoate (37.10%), cryptolepine (33.56%), quindoline (20.78%), 11-isopropylcryptolepine (5.16%), and hydroxycryptolepine (3.14%) were the active chemical ingredients in the drug with the concentrations of 18.64 ± 0.255, 16.85 ± 0.231, 10.42 ± 0.143, 2.56 ± 0.034, and 1.70 ± 0.023 *µ*g/mL, respectively. Administration of a single oral therapeutic dose (30 mL) of Mist Nibima corresponds to ingestion of 559.2 ± 7.662, 505.5 ± 6.930, 312.6 ± 4.285, 76.8 ± 1.028, and 51.0 ± 0.699 *µ*g of methyl cryptolepinoate, cryptolepine, quindoline, 11-isopropylcryptolepine, and hydroxycryptolepine, respectively. This translates into a corresponding daily dose of 1677.6 ± 22.986, 1516.5 ± 20.790, 937.8 ± 12.855, 230.4 ± 3.084, and 153.0 ± 2.097 *µ*g of methyl cryptolepinoate, cryptolepine, quindoline, 11-isopropylcryptolepine, and hydroxycryptolepine. These results could now serve as tools for authentication, standardization, and quality control of Mist Nibima to ensure its chemical and pharmacological consistency and safety.

## 1. Introduction

Mist Nibima is a decoction of the root of *Cryptolepis sanguinolenta* produced by the Center for Plant Medicine Research (CPMR), Mampong-Akuapim, Ghana. *C. sanguinolenta* (Lindl.) Schlechter is a climbing, twining, perennial shrub which is a member of the Apocynaceae family. The plant is locally known in Ghana as Nibima or Kadze in the Twi and Ewe dialects, respectively [1]. Mist Nibima is being sold and dispensed at the Center's Clinic as a key antimalaria herbal medicine in Ghana for over four decades. It is also used to treat other diseases such as candidiasis, upper respiratory tract infections, viral infections, urinary tract infections, diabetes, and lung diseases. Recently, there were reports of COVID-19 patients being cured after taking Mist Nibima. Furthermore, the Food and Drugs Authority (FDA), Ghana, and the National Medicine Regulatory Agency (NMRA), Ghana, approved the clinical trial of Mist Nibima (which is being sourced from CPMR) against COVID-19 to be undertaken by some scientist of the School of Public Health at the Kwame Nkrumah University of Science and Technology [2].

However, the chemical composition of Mist Nibima and a greater majority of herbal medicines on the global market are not known due to lack of standardization and chemical finger printing. This leads to variations in the chemical components, effectiveness, and/or periodic cases of toxicity due to toxic compounds which may be produced or absorbed by the plant since plants are variable raw materials [3, 4]. Additionally, medicinal plants and herbal medicinal products are also contaminated by assimilation of environmental contaminants such as toxic metals, pesticides, microbiological contaminants, foreign matter, and by-products of microorganisms as a result of improper postharvest handlings or during production and/or storage which makes the products deteriorate and unwholesome for consumption. These problems could be resolved by standardization, chemical finger print analysis, and quality control to help safeguard the uniformity, safety, and effectiveness of various batches of a particular herbal medicinal product [3, 4]. During standardization, various scientific assays and observations are employed to establish a set of standards or constant parameters or absolute qualitative and quantitative values for a set of inherent properties of the medicine which guarantees the assurance of quality, efficacy, safety, and reproducibility [5].

It had been reported that the most active antimalaria constituent of *C. sanguinolenta* root was the indoloquinoline alkaloid cryptolepine [6]. Although constituents in *C. sanguinolenta* root have been extensively revealed, the exact chemical ingredients and their quantities in Mist Nibima are not known. The aim of this study, therefore, is to employ UHPLC-MS to characterize and quantify the chemical ingredients in Mist Nibima and to also standardize the decoction with the alkaloid cryptolepine isolated from *C. sanguinolenta* root using the calibration curve method in addition to other phytochemical and physicochemical studies.

## 2. Materials and Methods

### 2.1. Chemicals and Reagents

Ethyl acetate was obtained from Merck KgaA, Darmstadt, Germany. Chloroform and petroleum ether (40–60°C) were procured from Fisher Scientific, Loughborough, and Park Scientific Northampton, UK, respectively. Ethanol (99%) was also purchased from Midland International, Ghana. Silica gel (normal phase for column chromatograph) and aluminum precoated normal phase TLC plates (6 F_254_) were also purchased from Merck KgaA, Darmstadt, Germany.

### 2.2. Isolation of Cryptolepine from the Root of *C. sanguinolenta*

The dried root of *C. sanguinolenta* was obtained from the stores of the Production Department of CPMR. It was pulverized into course powder and 2.0 kg was cold macerated with 5.0 L of 70% ethanol for 3 days. The extract was filtered and concentrated under low pressure in rotary evaporator to 0.5 L. Total alkaloid extract (13 g) was obtained after the crude extract (0.5 L) was basified with ammonia and exhaustively extracted with chloroform (0.5 L *x* 6) and dried into a solid in the rotary evaporator. The crude alkaloid extract (13 g) was chromatographed over a normal phase silica gel using petroleum ether-chloroform-ethanol in a gradient eluting manner to obtain dark purple needle crystals.

### 2.3. Mist Nibima

Three batches (NB. 20010; NB. 20011; NB. 20012) of Mist Nibima were obtained from the dispensary of the Cenetr for Plant Medicine Research, Mampong-Akuapim, for the study. The decoction was produced from the pulverized roots of *C. sanguinolenta* and packaged in 330 mL amber-colored plastic bottles. The drug was registered with the Food and Drugs Authority of Ghana with the number FDA/HD_1_.20–02086.

### 2.4. Evaluation of Physicochemical Properties of Mist Nibima

#### 2.4.1. Total Solid Residue (TSR)

Mist Nibima (10 mL) was measured into a ceramic evaporating dish with a known weight (Wo) and dried over a water bath in the hood. The dish was removed and dried in an oven to a constant weight at 70°C. It was then removed and allowed to cool to room temperature after which its weight (W_1_) was determined. TSR of the drug was calculated by relating the weight of the drug to its volume using the following formula:

(1) $$\begin{array}{c} \text{TSR}=\frac{\text{W}1-\text{Wo}}{\text{Volume of sample evaporated}}\times \;100\%\text{wv}. \end{array}$$

#### 2.4.2. Specific Gravity (SG)

Briefly, a dried empty gravity bottle with a known weight (*W*_o_) was fully filled with Mist Nibima and corked with the gravity bottle's stopper and weighed (*W*_1_) after being wiped dried. The drug in the gravity bottle was then discarded. The bottle was rinsed with distilled water. It was thereafter fully filled with distilled water, wiped dried, and weighed (*W*_2_). The SG of the sample was then calculated as the weight of the sample divided by the weight of water using the following formula:

(2) $$\begin{array}{c} \text{SG}=\;\frac{\text{W}1-\text{Wo}}{\text{W}2-\text{Wo}}. \end{array}$$

#### 2.4.3. pH

The pH of the drug was determined using a digital pH meter (PL-700 PV, Taiwan) with pH and temperature probes. The pH meter was first calibrated with standard pH buffers 4, 7, and 10 prior to being dipped into the drug in a 50 mL glass beaker.

#### 2.4.4. Color

The color of the drug was determined by observation with the eye and then matching it with colors on RAL Classic color chart (Ral color chart.pdf-e-paint.co.uk).

### 2.5. Identification of Phytochemical Constituents in Mist Nibima

Previously described methods [7] were employed to identify the classes of phytochemical constituents in Mist Nibima.

### 2.6. Quantification of Total Alkaloids in Mist Nibima

A revised method described by Kumatia et al. [8] which was earlier reported [9] was adopted to quantify the amount of alkaloid in Mist Nibima. Mist Nibima (100 mL) was evaporated to dryness on water bath, and 1.0 g of the dried sample was used for the test. The percentage of alkaloids in the drug was calculated with the following formula:

(3) $$\begin{array}{c} \text{Alkaloid}=\frac{\text{Weight of alkaloid}}{\text{Weight of sample}}\times 100\text{\%}. \end{array}$$

### 2.7. Identification of Cryptolepine Using UHPLC-MS

In order to establish the chemical identity of cryptolepine, 2.5 mg of the isolated compound was first taken through a similar process as described above in the UHPLC finger print evaluation of Mist Nibima. Additionally, the UHPLC was coupled with a Mass Spectrometer (Agilent Tripple Quadrupole MS) to determine the mass and fragmentation patterns of cryptolepine. The electron source ionization (ESI) (positive mode) was employed for fragmentation. The experimental conditions and the MS m/z scan range were as earlier reported [8]. The mobile phase employed in the UHPLC analysis (Sections 2.7–2.9) was 70% water and 30% methanol under isocratic conditions.

### 2.8. Preparation of a Standard Calibration Curve Using Cryptolepine

The quantity of cryptolepine in Nibima was determined by means of a calibration curve method using UHPLC. A standard calibration curve was generated using the pure cryptolepine isolated from *C. sanguinolenta* root. Cryptolepine (2.5 mg) was dissolved in 25 mL of HPLC grade methanol. Various concentrations of the solution (0.25, 0.5, 1.0, 2.0, 5.0, and 10.0 *µ*g/mL) were prepared from the original solution using serial dilution. The solutions were run on a UHPLC to obtain the response, retention time (RT), peak area, and peak height. The data acquired was processed using the MassHunter Quantitative Analysis software version B.07.00 (Agilent Technologies, USA) to produce a standard calibration curve by plotting the response against the concentration of cryptolepine. The calibration curve produced a linear regression equation in the form of

(4) $$\begin{array}{c} y=\text{ax}+b, \end{array}$$

where *y* is the peak area of cryptolepine in the crude medicine/sample, *a* and *b* are constants given by the calibration curve, and *x* is the concentration of the cryptolepine in the crude sample/medicine.

### 2.9. Determination of the Quantity of Cryptolepine in Mist Nibima

In order to determine the quantity of cryptolepine in Nibima, 100 mL of the drug was evaporated to dryness on water bath. The dried sample (2.0 g) was dissolved in 4 mL of methanol in a 50 mL centrifuge tube and thoroughly mixed with an electronic mixer (Votex mixer) for 5 minutes and then centrifuged at 3500 rpm. The top layer of the solution (2 mL) was pipetted into a pear-shaped flask and evaporated to dryness using a rotary evaporator. The residue was redissolved in 1 mL HPLC grade methanol and filtered with filter paper (mesh size = 0.45 um) into a GC vial and used for UHPLC analysis. The analysis was performed in duplicates using three batches of Nibima. Methanol was used as a blank. The data was processed using the Mass Hunter Quantitative Analysis software version B.07.00 (Agilent Technologies, USA). The quantity of cryptolepine in Mist Nibima was calculated using the linear regression equation [10] produced above from the calibration curve of the pure cryptolepine.

### 2.10. Chemical Fingerprinting and Identification of Compounds in Mist Nibima by UHPLC-MS

Chemical finger print analysis and identity of the compounds in Mist Nibima were evaluated according to a previously described method [8]. The dry sample of Mist Nibima (25 mg) was used.

### 2.11. Determination of the Percentage Compositions

The percentage composition of a given compound in the drug was calculated as follows:

(5) $$\begin{array}{c} \text{\% Composition of a compound}=\frac{\text{Area of the given compound in the drug}}{\text{Sum of the area of all the compounds in the drug}}\times 100\text{\%}. \end{array}$$

### 2.12. Determination of the Quantities of Other Compounds in Mist Nibima

The quantity of a given chemical ingredient/compound (QGCC), in *µ*g or mg, in Mist Nibima was calculated by relating the quantity of the cryptolepine (QCT), in *µ*g or mg, estimated in Mist Nibima and the percentage composition of cryptolepine (%CTP) in the drug and that of the given compound (%GCC) in the UHPLC chromatogram using the following formula:

(6) $$\begin{array}{c} \text{QGCC }\left(µ\text{g or mg}\right)=\frac{\text{\%GCC}\times \text{QCT}}{\text{\%CTP}}. \end{array}$$

## 3. Presentation of Data

Data generated from this study was analyzed using GraphPad Prism statistical software and presented as mean ± SEM.

## 4. Results and Discussion

### 4.1. Physicochemical and Organoleptic Properties of Mist Nibima

The results from the physicochemical and organoleptic tests are tabulated in Table 1.

Physicochemical properties of a drug are properties that relate to both physical and chemical characteristics of the drug/substance.

For decoctions, the physicochemical properties that are mostly evaluated are pH, specific gravity, total solid residue, and volume. Additionally, organoleptic parameters of a drug are those properties which are evaluated using the sense organs, e.g., taste, color, odor, texture, and dosage form. In this study, the organoleptic parameters evaluated for Mist Nibima include color, taste, and clarity. Individual drugs have unique physicochemical and organoleptic parameters which serve as fingerprints for standardization and quality control of future batches. Thus, this study has established standard physicochemical and organoleptic parameters for Mist Nibima (Table 1) which will be used as reference in the quality control of the subsequent batches of the drug.

### 4.2. Qualitative and Quantitative Phytochemical Constituents

The results of the qualitative and quantitative phytochemical analysis of Mist Nibima are tabulated in Tables 2 and 3.

The qualitative analysis indicates that the drug contains alkaloids saponins polyuronoids and free reducing sugars in all the three batches. The other six phytoconstituents were absent in the drug. Furthermore, the quantification tests also indicate that the alkaloids (23.71 ± 1.311%) content of the drug is 3 times the saponins (7.993 ± 0.067%) content (Table 3).

Besides, the physicochemical and organoleptic characteristics and qualitative and quantitative phytochemical constituent data of a herbal drug are the easiest and less expensive parameters used as standardization and quality control tools especially in a resource limited environments where modern instruments are too expensive to afford. Hence, these results also constitute a unique fingerprint for Mist Nibima which shall be used as a reference standard for quality control, standardization, and authentication of future batches of the drug.

### 4.3. Identification of Cryptolepine Using Chromatographic Profiles of Cryptolepine

**Figure 1:**
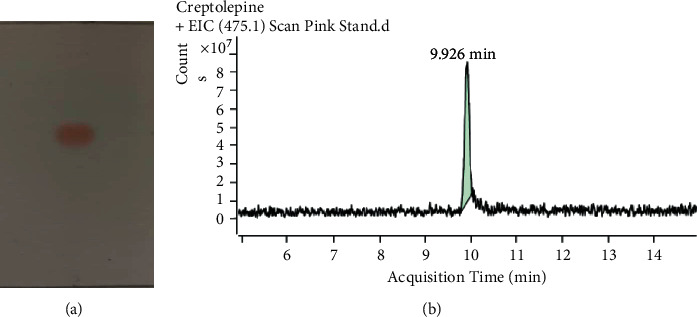
Chromatograms of cryptolepine isolated from *C. sanguinolenta* root. (a) TLC developed in chloroform/petroleum ether 9 : 1; (b) UHPLC chromatogram.

Cryptolepine was obtained as a purple needle crystal. The TLC and UHPLC chromatograms of cryptolepine are shown in Figures 1(a) and 1(b). The TLC chromatogram of the chloroform solution of cryptolepine produced a single pink spot when developed in 100% chloroform or chloroform/petroleum ether after the plate was dried in air for 10 mins and then immersed in 10% H_2_SO_4_ and heated at 100°C for 3 mins (Figure 1(a)). *R*_f_ = 0.5897 (chloroform) and 0.6111 (chloroform/petroleum ether 9 : 1). The purple color of cryptolepine and its former *R*_f_ value compare favorably with the color and *R*_f_ = 0.51 (chloroform/methanol/ammonia 90 : 10 : 1) reported for cryptolepine on a reverse phase TLC plate [11].

**Figure 2:**
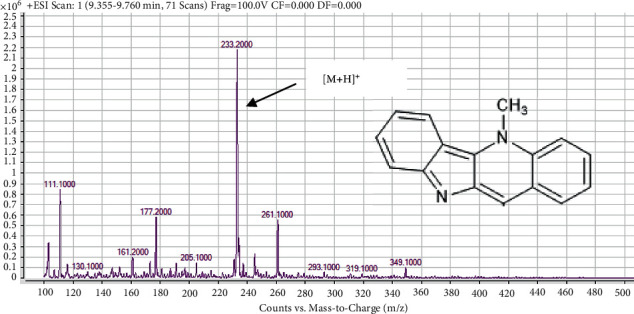
Mass spectrum and structure of cryptolepine isolated from *C. sanguinolenta* root.

The UHPLC chromatogram of cryptolepine also produced a single peak with a RT of 9.936 mins. The compound produced a single spot in two-solvent system in the TLC analysis and a single peak in the UHPLC chromatogram indicates that the isolated compound was pure. The mass spectrum and structure of the isolated compound from *C. sanguinolenta* root are shown in in Figure 2, which consists of a total of ten (10) peaks of ion fragments with mass to charge ratios ranging 103.30–363.20 (Figure 2). The ion peak at 233.20 revealed the [*M* + *H*]^+^ of C_16_H_12_N_2_ which is indicative of cryptolepine with a molecular mass (Mm of 232.28 g/mol) [12, 13]. The isolate was hence identified as cryptolepine based on its color, R*_f_*, and Mm.

### 4.4. Standard Calibration Curve of Cryptolepine

**Figure 3:**
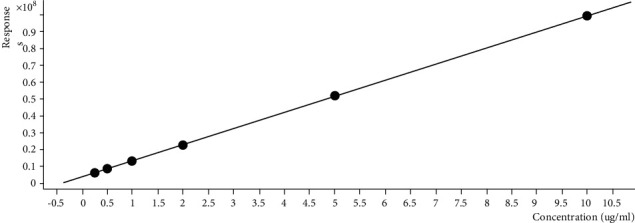
Standard calibration curve of cryptolepine (0.25–1000 *µ*g/mL).

Figure 3 shows the standard calibration curve generated using cryptolepine (0.25–1000 *µ*g/mL). The liner regression equation *y* = 9592831.280*x* + 3726013.583 was produced by the calibration curve with a regression coefficient (*R*^2^) of 0.99996109. The value of *R*^2^ obtained for the liner regression equation in this study was almost equal to 1 (100%). This indicates that the calibration process is linear and has a perfect fit during the experiment.

### 4.5. Quantity of Cryptolepine in Mist Nibima

The concentration, RT, transition, and area of cryptolepine in the various batches of Mist Nibima are shown in Table 4.

The quantity of cryptolepine in each batch of Mist Nibima was obtained by substitution of the area (*y*) in Table 4 into the linear regression equation (*y* = 9592831.280*x* + 3726013.583) generated from the calibration curve. The mean quantity of cryptolepine in the drug was then calculated to be 16.95 ± 0.231 *µ*g/mL. Each bottle (330 mL) of Mist Nibima, therefore, contains 5555 ± 55 *µ*g/mL (5.56 ± 0.055 mg/mL) of cryptolepine.

### 4.6. UHPLC Chemical Finger Print of Mist Nibima

**Figure 4:**
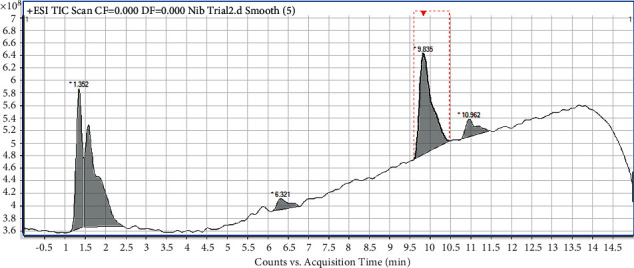
Chemical finger print of Nibima showing its UHPLC chromatogram.

The chemical finger print of Mist Nibima obtained using UHPLC is shown in Figure 4.

In order to develop a chemical fingerprint for the quality control of Mist Nibima and to also be sure that the alkaloid cryptolepine is part of the constituents extract into Mist Nibima by boiling *C. sanguinolenta* roots, UHPLC analysis of the drug was performed. The UHPLC chromatogram/chemical finger print of Mist Nibima shows the presence of five (5) distinct compounds in the drug with their respective RT. Cryptolepine is represented by the fourth peak with RT of 9.836 min which is similar to the RT (9.926 min) of pure cryptolepine (Figure 1). The RT of cryptolepine reduced by 0.021 min in the crude drug because the other constituents in the crude drug interfered with/blocked the movement of the cryptolepine through the UHPLC column.

Chemical fingerprinting is a critical tool used in quality control and standardization of herbal medicines to protect the homogeneity, safety, and efficacy of different batches of a herbal product/medicine [4]. A chemical fingerprint is a specific pattern which specifies the existence of various chemical markers in a sample. Chemical markers are defined as constituents or classes of constituents in a herbal medicinal product which are relevant for quality control purposes irrespective of their effectiveness as therapeutic principles [14]. The characteristic of the UHPLC chemical fingerprint described for Mist Nibima above (Figure 4) is therefore essential to safeguard the quality, authenticity, homogeneity, safety, and effectiveness of the future batches of the drug.

### 4.7. Identification of Compounds in Mist Nibima Using the MS Data

#### 4.7.1. Identification of Compound 1 (Peak 1) as Quindoline

**Figure 5:**
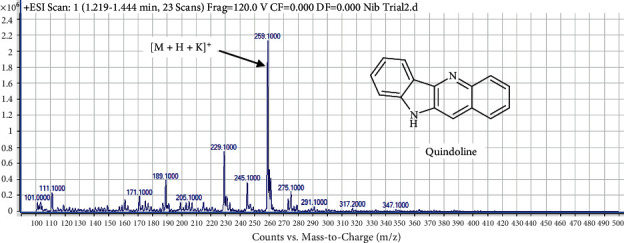
Mass spectrum and structure of compound 1 **(**quindoline) in Mist Nibima.

The structure and mass spectrum of compound 1 are shown in Figure 5. Compound 1 produced an adduct ion with 100% molecular ion peak m/*z* at 259.10 which is indicative of [*M* + *H* + K]^+^.

This represents the molecular mass of C_15_H_10_N_2_ which is indicative of quindoline with a molecular mass of 218.25 g/mol. Quindoline was previously isolated from (C) sanguinolenta root with *M*+ at m/*z* 218 (100%) by [13].

#### 4.7.2. Identification of Compound 2 (Peak 2) as Methyl Cryptolepinoate

**Figure 6:**
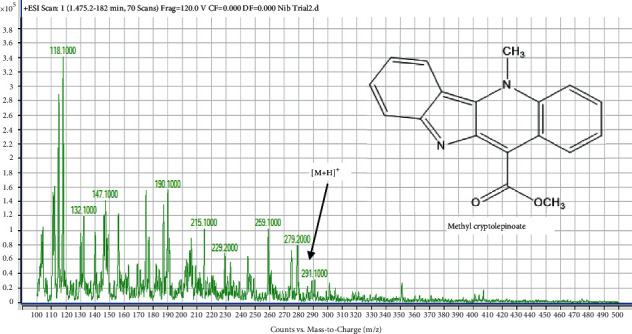
The mass spectrum of compound 2 **(**Methyl cryptolepinoate) in Mist Nibima.

The mass spectrum of the second compound in Mist Nibima is shown in Figure 6.

Compound 2 produced a fragment ion at m/*z* of 291.10 (0.6%) as the last peak in the spectrum which is indicative of [*M* + *H*]^+^. This represents the molecular formula of C_18_H_14_N_2_O_2_ with the molecular mass of 290.0 g/mol. This data agreed with the molecular weight of 290 and [*M* + *H*]^+^ of 291 reported for methyl cryptolepinoate [11]. Compound 2 was, therefore, identified as methyl cryptolepinoate (Figure 6).

#### 4.7.3. Identification of Compound 3 (Peak 3) as Hydroxycryptolepine

**Figure 7:**
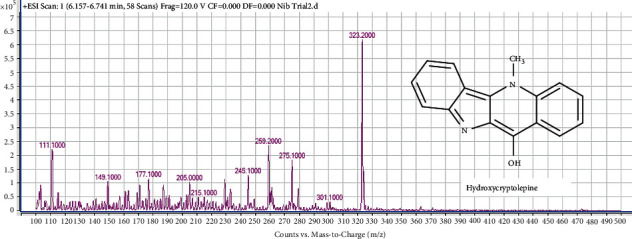
The mass spectrum and structure of compound 3 (hydroxycryptolepine) in Mist Nibima.

The mass spectrum of the second compound in Mist Nibima is shown in Figure 7.

Compound 3 produced at least 10 distinct peaks in its mass spectrum. The peak with m/*z* at 275 represents [*M* + 2H + Na]^+^ which is indicative of a sodiated adduct of C_16_H_12_N_2_0. This is indicative of hydroxycryptolepine which has a molecular mass (Mm) of 248 g/mol. The increase in Mm to 250 g/mol is due to the presence of the ^18^O isotope in hydroxyl group instead of ^16^O. Furthermore, the m/*z* observed at 259.2 in the spectrum is indicative of [*M* + 2H + Na]^+^ - CH_3_. These MS features observed for compound 3 confirmed that it is hydroxycryptolepine. Hydroxycryptolepine was isolated from *C. sanguinolenta* with [*M* + *H*]^+^ of 249 which led to Mm of 248 g/mol [11].

#### 4.7.4. Identification of Compound 4 (Peak 4) as Cryptolepine

**Figure 8:**
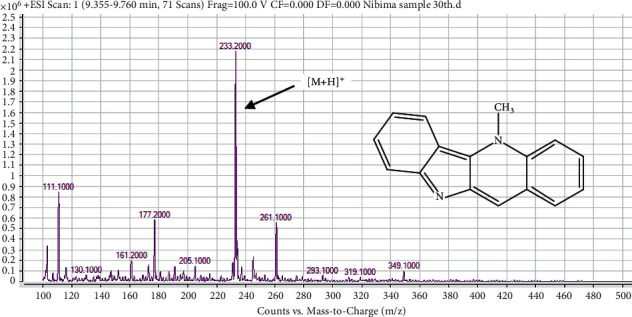
Mass spectrum and structure of compound 4 (cryptolepine) in Mist Nibima.

The mass spectrum of compound 4 is shown in Figure 8. Compound 4 showed similar mass spectral features which corresponds to those described for cryptolepine in Figure 2. Hence, compound 4 was identified as cryptolepine.

#### 4.7.5. Identification of Compound 5 (Peak 5) as 11-Isopropylcryptolepine

**Figure 9:**
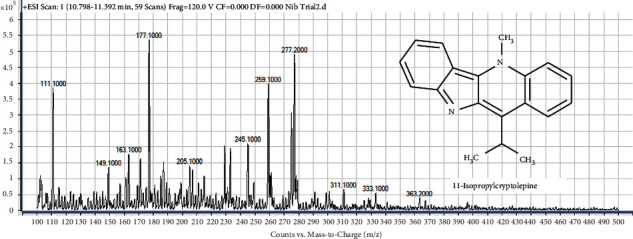
The mass spectrum of compound 5 (11-isopropylcryptolepine) in Mist Nibima.

The mass spectrum of the 5th compound in Nibima is shown in Figure 9. Compound 5 produced a molecular ion with m/*z* at 277. This represents the [*M* + *H*]+ of 11-isopropylcryptolepine which has a molar mass of 276 g/mol. Furthermore, the m/*z* at 259 and 245 represents [M -H-NH_3_]+ and [M-2H–2CH_3_]+, respectively, for 11-isopropylcryptolepine. Therefore, compound 5 was confirmed to be 11-isopropylcryptolepine [15].

### 4.8. Concentrations of Active Chemical Ingredients in Mist Nibima

The names, RT, and concentrations of the five active chemical ingredients/compounds identified in the herbal drug using the UHPLC-MS characterization are presented in Table 5.

The result indicates that methyl cryptolepinoate with 37.10% composition was the major component of Mist Nibima. This was followed by cryptolepine with 33.56%. The remaining three components followed in the decreasing order of quindoline (20.78%), 11-isopropylcryptolepine (5.16%), and hydroxycryptolepine (3.14%). Mist Nibima is a decoction (boiling water extract) of *C. sanguinolenta* root. Water is a very polar solvent due to its strong hydrogen bonds. Hence, the most polar compounds were to be extracted in larger quantities into the drug compared to the less polar ones. However, this principle is true for methyl cryptolepinoate, an ester which dissociate into a cation and anion when in water, but not for hydroxycryptolepine. This indicates that the amount of compound extracted by boiling water depended not only on the polarity of the compound, but also on the quantity of the compound present in the plant material. Hence, hydroxycryptolepine might be the smallest constituent produced by the batch of the plant used to produce the drug. The dose of Mist Nibima as indicated on the label is 30 mL to be taken orally, 3 times daily after meals. Based on the results in Table 5 above, a single administration of 30 mL of the drug is equivalent to ingestion of 559.2 ± 7.662, 505.5 ± 6.930, 312.6 ± 4.285, 76.8 ± 1.028, and 51.0 ± 0.699 *µ*g of methyl cryptolepinoate, cryptolepine, quindoline, 11-isopropylcryptolepine, and hydroxycryptolepine, respectively. This translates into a corresponding daily dose (30 mL three (3) times) of 1677.6 ± 22.986, 1516.5 ± 20.790, 937.8 ± 12.855, 230.4 ± 3.084, and 153.0 ± 2.097 *µ*g of methyl cryptolepinoate, cryptolepine, quindoline, 11-isopropylcryptolepine, and hydroxycryptolepine.

*C. sanguinolenta* has been widely studied for its biological activities and chemical constituents. Phytochemical investigations of organic extracts and fractions of the plant led to isolation of about eighteen indolo quinoline alkaloids in addition to three other derivatives of cryptolepine. They are cryptolepine, isocryptolepine, cryptosanguinolentine, neocryptolepine, cryptotackieine, quindoline, cryptospirolepine, cryptolepicarboline, cryptomisrine, 11-isopropylcryptolepine, cryptolepinone, biscryptolepine, hydroxycryptolepine, cryptoheptine cryptoquindoline, cryptolepinoic acid, methyl cryptolepinoate, and ethyl cryptolepinoate [6, 11, 13, 15–20]. However, the exact chemical constituents and their concentration in the herbal drugs/preparations of *C. sanguinolenta* including Mist Nibima were not known. This study has chromatographically and spectroscopically characterized the individual chemical compounds in Mist Nibima and also determined their concentration for the first time.

The UHPLC chemical finger printing analysis has shown that Mist Nibima contains five compounds out of about eighteen alkaloids previously isolated from the plant, since the chemical fingerprint has shown that the drug contains five compounds (five peaks) with specific RTs. It is now possible to discriminate contaminants in any future batch of the drug or be able to tell if an additional peak detected in any future batch of Mist Nibima is being produced by the plant or is an adulterant by using this chemical fingerprint as a reference standard. The chemical finger print chromatogram obtained for Mist Nibima could, therefore, serve as reference standard in determination of adulteration and substandard batches of the drug in future productions.

Furthermore, knowing the standard concentration of a drug makes it possible to distinguish between a substandard batch with significantly lower concentration of the active ingredients or a batch with significantly high concentrations of the active ingredients which may be toxic. These situations may arise as a result of lapses in the raw material sourcing and the manufacturing process. Since the respective compounds and their standard concentrations in Mist Nibima are now known, the necessary adjustments can be made to any batch which does not meet these standard concentrations of the active ingredients in order to bring it to the level of the reference concentrations of its active ingredients. For instance, the concentration of active ingredients in a lower concentrated batch of a herbal medicine can be increased to the standard levels by evaporations under controlled heat to a required volume or addition of a calculated amount of a high concentrated sample, whereas a batch with higher concentration of active ingredients of the drug can be diluted with calculated amount of water or low concentrated sample to reduce it to the standard concentration.

## 5. Conclusions

The present study has established physicochemical, organoleptic, qualitative, and quantitative phytochemical standardization and quality control parameters for Mist Nibima, a herbal decoction used for the treatment of malaria, COVID-19, and many other diseases. Five indole alkaloids, namely, quindoline, methyl cryptolepinoate, hydroxycryptolepine, cryptolepine, and 11-isopropylcryptolepine, and their corresponding concentrations have also been identified as the active chemical constituents in Mist Nibima for the first time. These results will be used as tools for authentication, standardization, and quality control of this essential herbal medicine to ensure its consistent chemical and pharmacological characteristics and safety.

## Figures and Tables

**Table 1:** Physicochemical and organoleptic properties of Mist Nibima.

Physicochemical parameters		Organoleptic properties	
Parameter	Mean ± SEM	Property	Nature
pH @ 28.3°C	6.10 ± 0.08	Color	Broom yellow (RAL 1032)
Total solid residue	5.34 ± 0.27%w/v	Taste	Very bitter
Specific gravity	1.0099 ± 0.0000	Clarity	Cloudy

**Table 2:** Results of the qualitative phytochemical analysis of Mist Nibima.

Test	Result	Test	Result
Alkaloids	Present	Flavonoids	Absent
Saponins	Present	Antracenosides	Absent
Polyuronoids	Present	Phenolic compounds	Absent
Free reducing sugars	Present	Triterpenes	Absent
Phytosterols	Absent	Cyanogenic glycosides	Absent

**Table 3:** Percentage compositions of saponins and alkaloids in Mist Nibima.

Phytochemical constituent	Quantity (%) in Nibima
Alkaloids	23.71 ± 1.311
Saponins	7.993 ± 0.067

**Table 4:** Concentration, RT, transition, and area of cryptolepine obtained in three batches of Mist Nibima and the blank.

Sample label	RT	Transition	Area	Height	Final conc. (*µ*/mL)
NB.1A 20010	9.736	233.3	167476917	9026126	17
NB.1B 20010	9.744	233.3	171346787	9031102	17
NB.2A 20011	9.746	233.3	167070077	8967518	17
NB.2B 20011	9.741	233.3	165832143	8944253	17
NB.3A 20012	9.739	233.3	165832143	8968638	17
NB.3B 20012	9.744	233.3	158318774	8822598	16
Blank	9.736	233.3	—	—	None detected

**Table 5:** Names of compounds/active ingredients identified in Mist Nibima and their RTs, peak areas, percentage compositions, and concentrations in Mist Nibima.

Peak	Proposed compound	RT (min)	Area	Composition in Mist Nibima (%) (*µ*g/mL) (mg/330 mL bottle)		
1	Quindoline	1.352	2070555301.3	20.78	10.42 ± 0.143	3.44 ± 0.047
2	Methyl cryptolepinoate	1.577	3697465123.3	37.10	18.64 ± 0.255	6.15 ± 0.084
3	Hydroxycryptolepine	6.321	339811726.26	3.41	1.70 ± 0.023	0.56 ± 0.011
4	Cryptolepine	9.835	3344237823.9	33.56	16.85 ± 0.231	5.56 ± 0.076
5	11-Isopropylcryptolepine	10.962	514102921.06	5.16	2.56 ± 0.034	0.85 ± 0.007

## Data Availability

The data generated and analyzed in this study can be obtained from the corresponding author upon reasonable request.
